# Exercise‐induced potentiation of the acute hypoxic ventilatory response: Neural mechanisms and implications for cerebral blood flow

**DOI:** 10.1113/EP091330

**Published:** 2024-03-05

**Authors:** Diogo M. Oliveira, Anas Rashid, Patrice Brassard, Bruno M. Silva

**Affiliations:** ^1^ Postgraduate Program in Translational Medicine, Department of Medicine Paulista School of Medicine (EPM) Federal University of São Paulo (UNIFESP) São Paulo Brazil; ^2^ Pulmonary Function and Clinical Exercise Physiology Unit (SEFICE), Division of Pneumology, Department of Medicine, Paulista School of Medicine (EPM) Federal University of São Paulo (UNIFESP) São Paulo Brazil; ^3^ Department of Kinesiology, Faculty of Medicine Université Laval Québec City QC Canada; ^4^ Research Centre of the Institut Universitaire de Cardiologie et de Pneumologie de Québec Québec QC Canada; ^5^ Department of Physiology, Paulista School of Medicine (EPM) Federal University of São Paulo (UNIFESP) São Paulo Brazil

**Keywords:** brain, breathing, exertion, hypocapnia, hypoxia, reflex, synergism

## Abstract

A given dose of hypoxia causes a greater increase in pulmonary ventilation during physical exercise than during rest, representing an exercise‐induced potentiation of the acute hypoxic ventilatory response (HVR). This phenomenon occurs independently from hypoxic blood entering the contracting skeletal muscle circulation or metabolic byproducts leaving skeletal muscles, supporting the contention that neural mechanisms per se can mediate the HVR when humoral mechanisms are not at play. However, multiple neural mechanisms might be interacting intricately. First, we discuss the neural mechanisms involved in the ventilatory response to hypoxic exercise and their potential interactions. Current evidence does not support an interaction between the carotid chemoreflex and central command. In contrast, findings from some studies support synergistic interactions between the carotid chemoreflex and the muscle mechano‐ and metaboreflexes. Second, we propose hypotheses about potential mechanisms underlying neural interactions, including spatial and temporal summation of afferent signals into the medulla, short‐term potentiation and sympathetically induced activation of the carotid chemoreceptors. Lastly, we ponder how exercise‐induced potentiation of the HVR results in hyperventilation‐induced hypocapnia, which influences cerebral blood flow regulation, with multifaceted potential consequences, including deleterious (increased central fatigue and impaired cognitive performance), inert (unchanged exercise) and beneficial effects (protection against excessive cerebral perfusion).

## Abstract (highlights)

- **What is the topic of this review?**
  What neural mechanisms are involved in the ventilatory response to hypoxic exercise and do they interact? What are the mechanisms underlying neural interactions during hypoxic exercise?
- **What advances does it highlight?**
  Current evidence does not support an interaction between the carotid chemoreflex and central command. In contrast, findings from some studies support synergistic interactions between the carotid chemoreflex and the muscle mechano‐ and metaboreflexes. Medullary signal summation, short‐term potentiation and sympathetic carotid chemoreceptor activation probably contribute to potentiation of the hypoxic ventilatory response during exercise, impacting cerebral blood flow and oxygenation via hyperventilation‐induced hypocapnia.

## 1 INTRODUCTION

An acute reduction in oxygen (O_2_) availability in the inspired air challenges the maintenance of homeostasis, triggering multiple counter‐regulatory responses, such as increased pulmonary ventilation (Pamenter & Powell, 2016). In humans, the compensatory increase in pulmonary ventilation is mediated predominantly by specialized chemoreceptor cells located in the carotid bodies (Timmers et al., 2003). This notion is supported by evidence that patients who have undergone bilateral removal of the carotid bodies show a minor or absent hypoxic ventilatory response (HVR) (Timmers et al., 2003). Interestingly, a given dose of hypoxia elicits a greater increase in pulmonary ventilation during physical exercise than during rest (Regensteiner et al., 1988; Weil et al., 1972), indicating an exercise‐induced potentiation of the HVR. This potentiation is ubiquitous in healthy young humans, because it has been observed during varied types of exercise, including static (de Oliveira et al., 2023) and dynamic skeletal muscle contractions (Fregosi & Seals, 1993; Regensteiner et al., 1988; Weil et al., 1972) of small (de Oliveira et al., 2023; Fregosi & Seals, 1993) and large muscle groups (Fukuoka et al., 2003; Regensteiner et al., 1988) contracting at submaximal (de Oliveira et al., 2023; Fukuoka et al., 2003; Regensteiner et al., 1988; Weil et al., 1972) and maximal intensity (Fregosi & Seals, 1993).

The humoral and neural mechanisms involved in exercise‐induced potentiation of the HVR and the manner in which they operate remain incompletely understood. However, the potentiation occurs independently from hypoxic blood entering the contracting skeletal muscle circulation or metabolic byproducts leaving the skeletal muscles (Fregosi & Seals, 1993), supporting the contention that neural mechanisms per se can mediate potentiation of the HVR when humoral mechanisms are not at play. In this sense, integrative physiology studies performed in healthy humans have provided new insights regarding the neural side of the phenomenon (de Oliveira et al., 2023; Machado et al., 2020; Silva et al., 2018; Wan et al., 2020). Additionally, a study suggests that neural interaction between the carotid chemoreflex and the muscle metaboreflex contributes to the exaggerated ventilatory response to exercise in patients with heart failure with a reduced ejection fraction (HFrEF) (Machado et al., 2020), and a similar phenomenon could occur in other diseases presenting enhanced responses either to the isolated carotid chemoreflex or to muscle metaboreflex activation. The consequence of that could be a reduction in the cerebral blood flow response to exercise attributable to hyperventilation‐induced hypocapnia.

First, we discuss interactions between neural mechanisms involved in the ventilatory response to hypoxic exercise. Second, we propose hypotheses about the potential mechanisms underlying neural interactions, which might be beneficial to advance further investigations. Lastly, we ponder how exercise‐induced HVR potentiation results in hyperventilation‐induced hypocapnia, which influences cerebral blood flow regulation, with potential multifaceted implications.

## 2 INTERACTION BETWEEN CAROTID CHEMOREFLEX AND CENTRAL COMMAND

The notion that exercise‐induced HVR potentiation occurs during different types of exercise of varied intensities suggests that activation of central command, which is always present during active exercise, might interact with hypoxia‐induced carotid chemoreflex activation. However, to our knowledge, only one study has investigated this hypothesis (Pandit & Robbins, 1994). The HVR was quantified at rest, during electrically induced bilateral quadriceps contractions in an attempt to avoid activation of central command, and during voluntary exercise matched either to the electrically induced exercise external workload or to metabolic rate. The end‐tidal carbon dioxide (CO_2_) was clamped 1–2 Torr above the resting level to avoid an influence of CO_2_ on the peripheral and central chemoreflex drive. All types of exercise increased the HVR in comparison to rest. The HVR during the electrically induced exercise and metabolic rate‐matched voluntary exercise was similar, and both HVRs were greater than the one induced by voluntary exercise matched for external workload. Therefore, these results indicate that activation of brain areas involved in voluntary exercise is not obligatory for the occurrence of exercise‐induced HVR potentiation. However, the electrical stimulation method does not exclude the activation of motor planning areas (Williamson et al., 2002) and spinal cord pattern generators (Hérent et al., 2023), which are also part of the central command. Thus, future studies should revisit the interaction between the carotid chemoreflex and central command using alternative methods to manipulate central command.

## 3 INTERACTION BETWEEN CAROTID CHEMOREFLEX AND EXERCISE PRESSOR REFLEX

**FIGURE 1:**
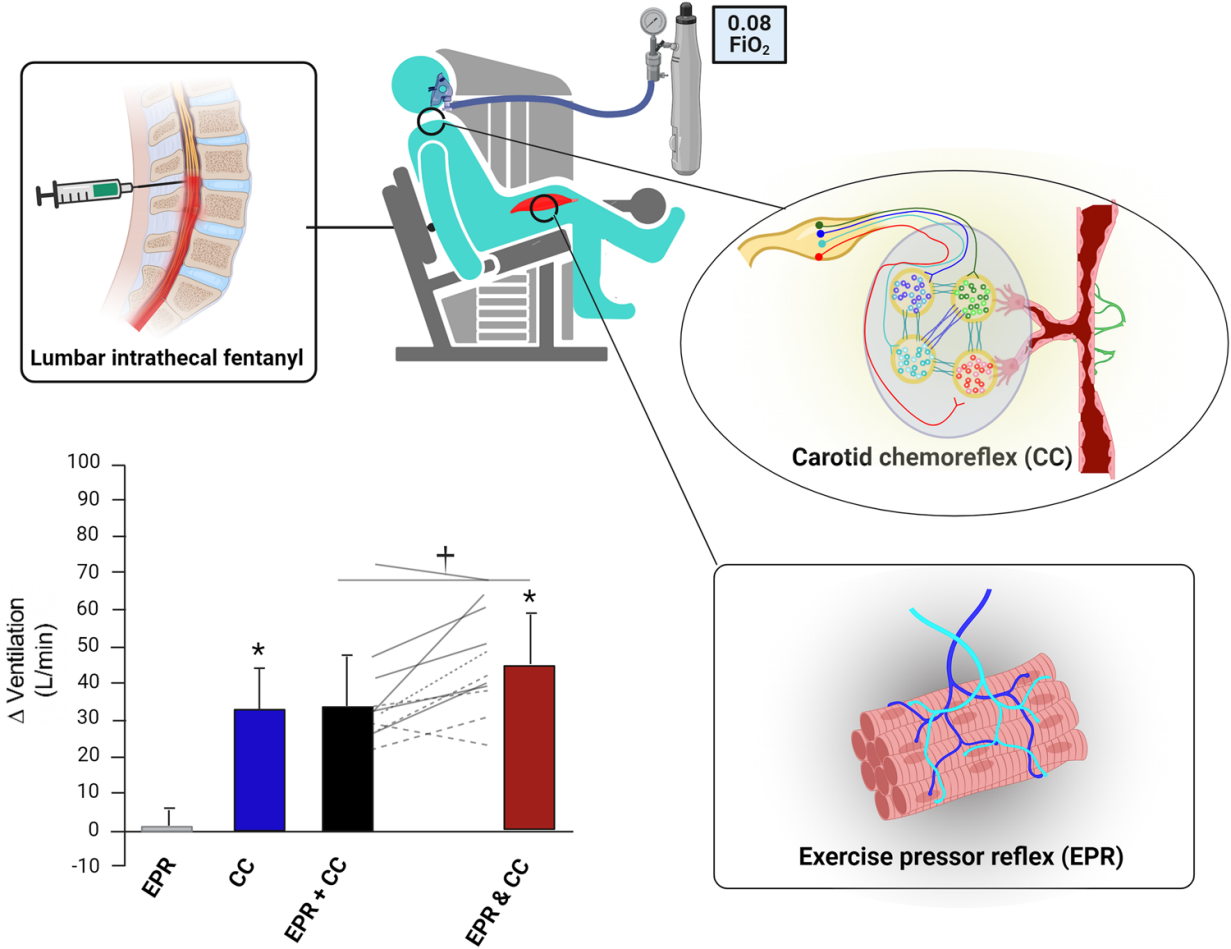
Illustration of a study that supports an interaction between the carotid chemoreflex (CC) and the exercise pressor reflex (EPR). The CC was activated by isocapnic hypoxia [inspired oxygen fraction (FiO_2_) = 0.08] or maintained at its tonic level by isocapnic normoxia. The EPR was attenuated by lumbar intrathecal fentanyl injection or maintained at its tonic level by no injection. The data represent the change in pulmonary ventilation as a result of activation of each reflex separately (i.e., EPR or CC), the sum of separate activation of the reflexes (i.e., EPR + CC) and coactivation of the reflexes (EPR and CC). Note that the coactivation effect was greater than the summed effect. ^*^ *P* < 0.05 versus zero; ^†^ *P* < 0.05 EPR and CC versus EPR + CC. Data reproduced from Wan et al. (2020).

The findings from one study firmly support an interaction between hypoxia‐induced activation of the carotid chemoreflex and exercise‐induced activation of muscle afferents (i.e., the exercise pressor reflex) in the regulation of pulmonary ventilation during hypoxic exercise (Wan et al., 2020; Figure 1). During one experimental session, the study participants received a lumbar intrathecal injection of fentanyl to attenuate the afferent feedback from type III and IV muscle afferents (i.e., exercise pressor reflex attenuation) without influencing motor control. During another session, no injection was delivered (i.e., exercise pressor reflex activation). Then, participants performed constant‐load rhythmic knee extension with the right limb under isocapnic normoxia (i.e., tonic carotid chemoreflex activity) and isocapnic hypoxia (i.e., carotid chemoreflex activation). The normoxic exercise under fentanyl served as a reference to interpret the effect of separate and combined activation of the reflexes on pulmonary ventilation. The authors reported that carotid chemoreflex–exercise pressor reflex coactivation elicited a greater ventilatory response than the sum of the isolated responses elicited by each separate reflex activation, indicating the presence of a synergistic or hyperadditive interaction.

Although this study using fentanyl provides strong evidence supporting a carotid chemoreflex–muscle reflex interaction (Wan et al., 2020), the muscle reflex manipulation did not allow separation of the roles played by each type of muscle reflex, and the muscle reflex is composed of the mechanoreflex and the metaboreflex. The exercise‐induced potentiation of the HVR can occur during moderate‐intensity exercise (Weil et al., 1972), in which, theoretically, the muscle mechanoreflex is activated whereas the muscle metaboreflex is not (Kaufman et al., 1983), suggesting that the carotid chemoreflex might interact with the muscle mechanoreflex. Furthermore, the exercise‐induced HVR potentiation increases with exercise intensity (Weil et al., 1972). As the build‐up of metabolites within skeletal muscles depends on exercise intensity (Bendahan et al., 1996), leading to activation of metabolically sensitive muscle afferents (Kaufman et al., 1983), it is plausible that the interaction of hypoxia‐induced carotid chemoreflex activation and exercise‐induced muscle metaboreflex activation might also contribute to mediating the exercise‐induced HVR potentiation.

**FIGURE 2:**
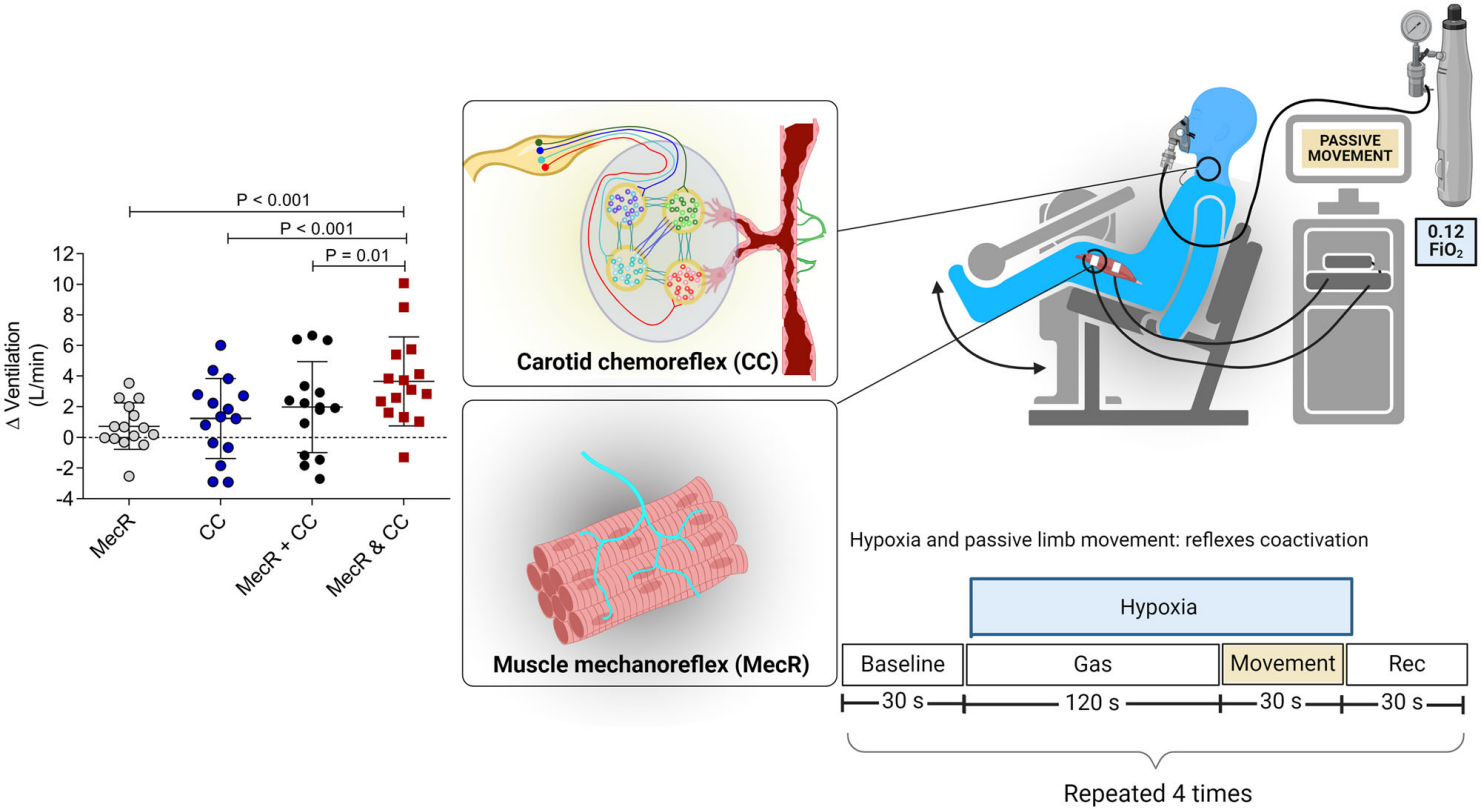
Illustration of a study that investigated interaction between the carotid chemoreflex (CC) and the muscle mechanoreflex (MecR). The CC was activated by isocapnic hypoxia [inspired oxygen fraction (FiO_2_) = 0.12] or maintained at its tonic level by isocapnic normoxia. The MecR was activated by passive limb movement or not activated, by resting. The data represent the change in pulmonary ventilation as a result of activation of each reflex separately (i.e., MecR or CC), the sum of activation the separate reflexes (i.e., MecR + CC) and coactivation of the reflexes (MecR and CC). Note that the coactivation effect was greater than the summed effect. Abbreviation: Rec, recovery. Data reproduced from Silva et al. (2018).

A study by our group investigated the carotid chemoreflex–muscle mechanoreflex interaction (Silva et al., 2018; Figure 2). We compared ventilatory responses to separate hypoxia‐induced carotid chemoreflex activation and passive limb movement‐induced muscle mechanoreflex activation, with the ventilatory response to coactivation of reflexes elicited by hypoxic passive limb movement. Experiments were conducted under isocapnia. The passive limb movement was performed by flexing and extending the dominant knee in a standardized manner using an isokinetic dynamometer. The absence of active contractions was verified via surface EMG recording of the quadriceps muscle. It was found that the ventilatory response to coactivation of the reflexes was greater than the summed ventilatory responses to separate activation of the reflexes, providing evidence for a synergistic or hyperadditive interaction between the carotid chemoreflex and the muscle mechanoreflex.

Some studies included hypoxic postexercise circulatory occlusion (PECO; i.e., coactivation of the carotid chemoreflex and muscle metaboreflex), hypoxic rest (i.e., carotid chemoreflex activation) and normoxic PECO (i.e., muscle metaboreflex activation), in an attempt to investigate the isolated and combined effect of activation of the carotid chemoreflex and muscle metaboreflex (Edgell & Stickland, 2014; Gujic et al., 2007; Houssiere et al., 2005). However, either the summed effect was not calculated (Gujic et al., 2007; Houssiere et al., 2005), which did not allow interpretation of the nature of integration of the reflexes (i.e., hyperadditive, additive or hypoadditive), or, paradoxically, coactivation and summed effects produced similar results (i.e., additive integration; Edgell & Stickland, 2014), indicating no interaction between the reflexes.

**FIGURE 3:**
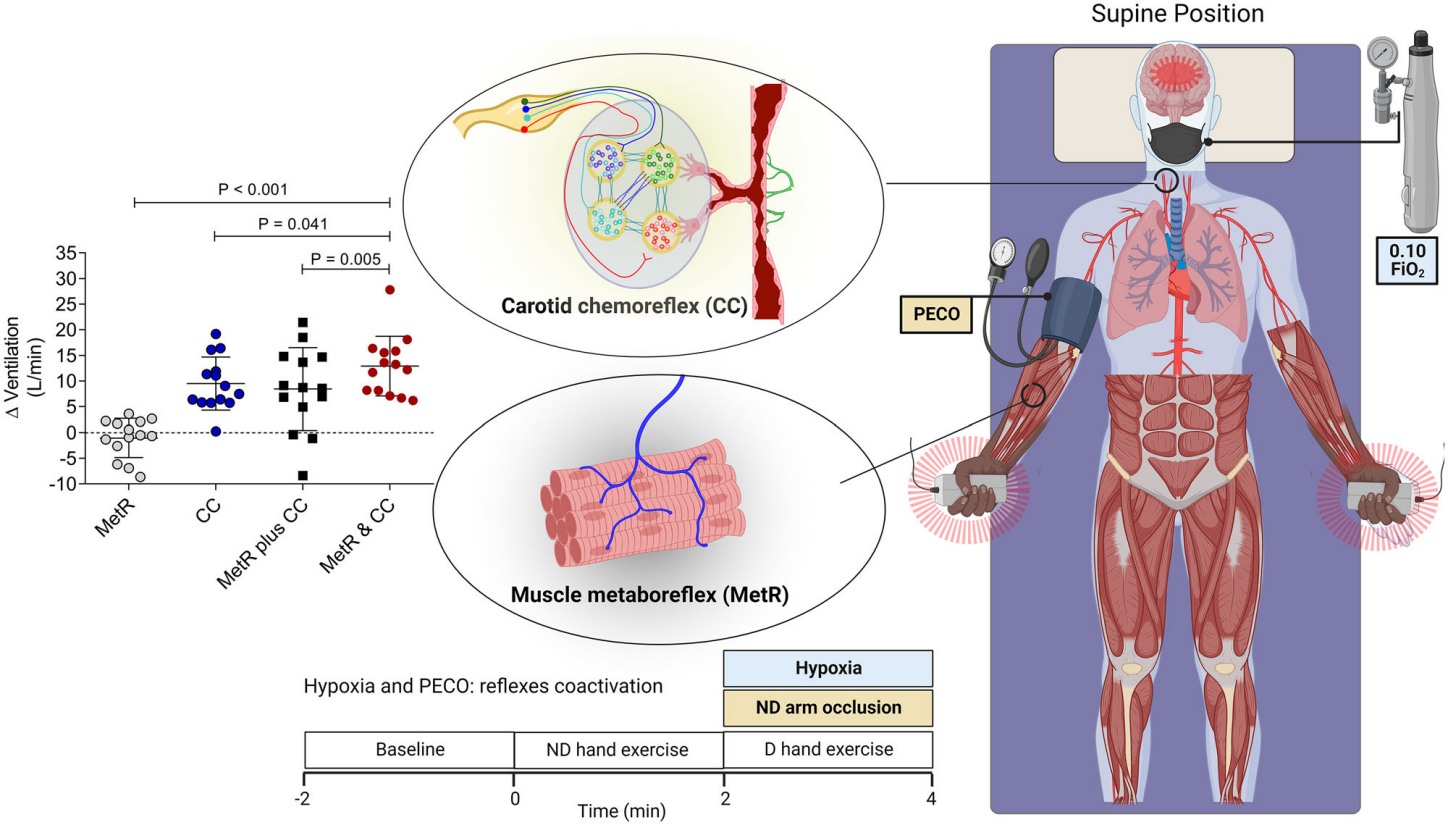
Illustration of a study that investigated the interaction between the carotid chemoreflex (CC) and the muscle metaboreflex (MetR). The CC was activated by isocapnic hypoxia [inspired oxygen fraction (FiO_2_) = 0.10] or maintained at its tonic level by isocapnic normoxia. The MetR was activated by postexercise circulatory occlusion (PECO) in one arm during exercise with the contralateral hand, or not inactivated by not using occlusion in one arm during exercise with the contralateral hand. The data represent the change in pulmonary ventilation as a result of activation of each reflex separately (i.e., MetR or CC), the sum of activation of the separate reflexes (i.e., MetR + CC) and coactivation of the reflexes (MetR and CC). Note that the coactivation effect was greater than the summed effect. Abbreviations: D, dominant; ND, non‐dominant. Data reproduced from de Oliveira et al. (2023).

We recently revisited the interaction between the carotid chemoreflex and the muscle metaboreflex using hypoxia to activate the carotid chemoreflex and PECO on an arm, while the other arm performed static handgrip, to activate the muscle metaboreflex in healthy adults (de Oliveira et al., 2023; Figure 3). The PECO was performed during exercise because it typically does not influence pulmonary ventilation in healthy adults when used at rest (Lam et al., 2019). In addition, the end‐tidal partial pressure of CO_2_ was clamped 1–2 Torr above the resting level to avoid bias related to varied CO_2_ levels between conditions (Bruce & White, 2012). We reported that simultaneous carotid chemoreflex–muscle metaboreflex activation yielded a greater ventilatory response than the sum of separate carotid chemoreflex and muscle metaboreflex responses, thereby providing evidence for a synergistic or hyperadditive interaction between the carotid chemoreflex and the muscle metaboreflex in healthy adults.

It is noteworthy that we found comparable findings in patients with HFrEF (Machado et al., 2020). In this case, we compared the pulmonary ventilation during recovery from cycling exercise among four conditions: (1) inspired oxygen fraction (FiO_2_) = 0.21 (i.e., tonic carotid chemoreflex activity) with circulation in the legs free (i.e., inactive muscle metaboreflex); (2) FiO_2_ = 1.0 (i.e., reduced carotid chemoreflex activity) with circulation in the legs arrested (i.e., muscle metaboreflex activation); (3) FiO_2_ = 0.21 (i.e., tonic carotid chemoreflex activity) with circulation in the legs arrested (i.e., muscle metaboreflex activation); or (4) FiO_2_ = 1.0 (reduced carotid chemoreflex activity) with circulation in the legs free (inactive muscle metaboreflex) as control. The summed effect of activation of the separate reflexes on pulmonary ventilation was similar to the control response. However, the pulmonary ventilation response was greater than the control response during simultaneous activation of the reflexes. The results indicate that the prevailing tonic carotid chemoreceptor activity during normoxia interacted synergistically with muscle metaboreceptor inputs, and such interaction could contribute to the poor prognostic hyperventilation response during normoxic exercise in HFrEF (Guazzi et al., 2005). The interaction in normoxia could be attributed to the low cardiac output in HFrEF, which increases the tonic activity of carotid chemoreceptors via reduced perfusion of the carotid bodies (Li et al., 2006) and is linked to exaggerated activation of muscle metaboreceptors (Wang et al., 2010). Importantly, other patients also present with enhanced responses to separate activation of the carotid chemoreceptors and muscle metaboreflex and with excessive breathing drive during exercise, such as patients with pulmonary arterial hypertension (Paula‐Ribeiro et al., 2021, 2019; Plunkett et al., 2024; Sayegh et al., 2021), obstructive sleep apnoea (Hargens et al., 2009; Narkiewicz et al., 1999) and chronic obstructive pulmonary disease (Aranda et al., 2024; Stickland et al., 2016). Therefore, a carotid chemoreflex–muscle reflex interaction might also operate and play a relevant role during exercise in diseases other than HFrEF.

## 4 CENTRAL MECHANISMS UNDERLYING SYNERGISTIC INTERACTIONS BETWEEN NEURAL MECHANISMS

**FIGURE 4:**
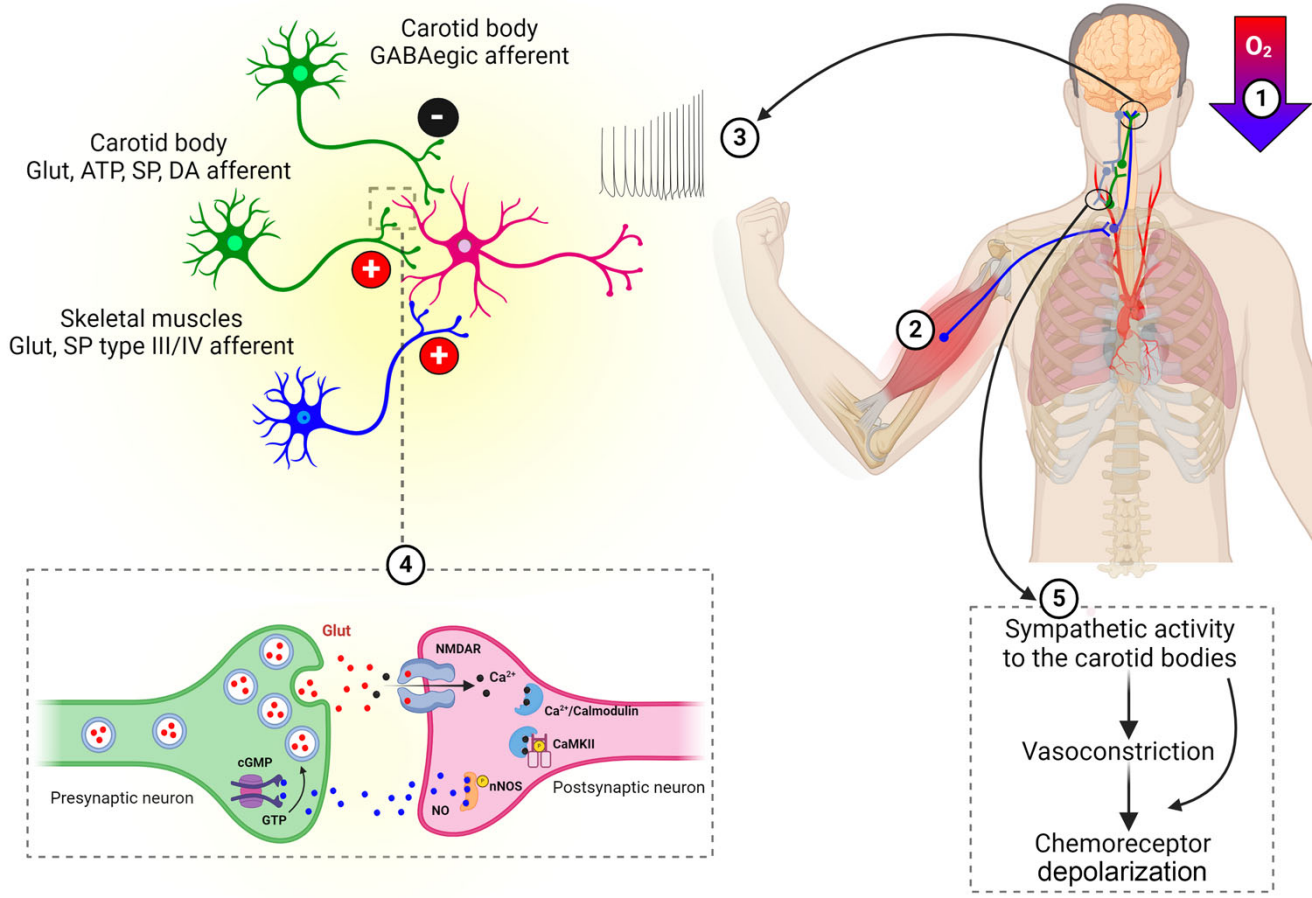
Potential mechanisms underlying synergistic interactions. (1) A decrease in arterial partial pressure of O_2_ is detected predominantly by peripheral chemoreceptors in the carotid bodies, which send afferent signals to the nucleus tractus solitarii (NTS). (2) Skeletal muscle contractions activate type III/IV muscle afferent fibres, which also synapse with NTS neurons. (3) Afferent inputs from both sources might converge upon similar brainstem neurons, amplifying the depolarization frequency of brainstem neurons. (4) Glutamate (Glut) release from presynaptic neurons might activate a cascade pathway in postsynaptic neurons, enhancing Glut release by presynaptic neurons as a positive feedback loop, thereby generating short‐term potentiation. (5) Increased sympathetic activity can depolarize chemoreceptor cells directly. Additionally, sympathetic activation to the carotid bodies can elicit local vasoconstriction, indirectly resulting in chemoreceptor depolarization. Both pathways can further increase afferent signalling by the carotid bodies, thus operating as a positive feedback loop. Abbreviations: CaMKII, calcium/calmodulin‐stimulated protein kinase II; cGMP, cyclic guanosine monophosphate; DA, dopamine; GTP, guanosine triphosphate; NMDAR, *N*‐methyl‐d‐aspartate receptor; nNOS, neuronal nitric oxide synthase; SP, substance P.

An interaction between the carotid chemoreflex and exercise‐related neural signalling might occur either in the CNS or in the periphery. The central interaction might occur owing to temporal and spatial summation of afferent signalling into common neurons (Smith et al., 1990; Figure 4). A potential site of interaction is the nucleus tractus solitarii (NTS), where afferents from carotid chemoreceptors (Lindefors et al., 1986; Teppema et al., 1997) and muscle afferents initially synapse in the brainstem (Potts & Waldrop, 2005). Carotid chemoreceptor afferents release the excitatory neurotransmitters glutamate, substance P, ATP and dopamine and the inhibitory neurotransmitter GABA onto NTS neurons (Cutsforth‐Gregory & Benarroch, 2017; Lindefors et al., 1986). The muscle afferents release the excitatory neurotransmitters glutamate and substance P onto NTS neurons (Potts et al., 2007, 1999). The combined effect of hypoxia and exercise on neurotransmitter release onto NTS neurons has not been investigated. However, combined activation of baroreceptors and muscle receptors results in a higher substance P concentration in the extracellular space of NTS neurons than the sum of isolated activation of baroreceptors and muscle receptors, suggesting a convergence of afferent inputs, which facilitates the release of substance P (Lindefors et al., 1986). If a similar effect results from the activation of carotid chemoreceptors and muscle receptors, the net effect on the membrane potential of NTS neurons will depend ultimately on the sum of excitatory and inhibitory postsynaptic potentials, with the combined activation of carotid and muscle afferents most probably resulting in an increased discharge frequency of NTS neurons.

Convergent inputs might also occur in sites other than the NTS. C‐Fos immunohistochemistry showed that the retrotrapezoid/parafacial region was activated after both hypoxia (Takakura et al., 2006; Teppema et al., 1997) and running exercise (Barna et al., 2012, 2014) in awake rats. Likewise, single‐unit recordings showed an increased discharge frequency of retrotrapezoid nucleus (RTN) neurons during hypoxia and femoral or sciatic (Kanbar et al., 2016) nerve stimulation (i.e., exercise simulation) in anaesthetized rats. The RTN neurons make extensive projections to the entire ventral respiratory column (Rosin et al., 2006), where central respiratory pattern generators and premotor respiratory neurons reside. Consequently, bilateral RTN inhibition in anaesthetized rats via muscimol microinjection abolishes phrenic nerve discharge during hypoxia (Takakura et al., 2006), and systemic clozapine *N*‐oxide infusion to inhibit neurons transduced to express HM4D receptors in the parafacial region (which includes the RTN) reduces the ventilatory response to electrical stimulation of the sciatic or femoral nerve in anaesthetized spontaneously breathing rats (Korsak et al., 2018). In sum, current evidence supports that RTN/parafacial neurons are activated by hypoxia and exercise and that they contribute to hypoxia and exercise ventilatory responses. Therefore, this is a potential site for temporal and spatial input summation during the combination of hypoxia and exercise exposure.

In addition to temporal and spatial summation, another central mechanism that could be involved in the exercise‐induced potentiation of the HVR is short‐term potentiation, because a delayed decrease in pulmonary ventilation has been observed after hypoxic voluntary exercise (Fregosi & Seals, 1993) and hypoxic passive limb movement in humans (Silva et al., 2018). Short‐term potentiation represents an increased response that outlasts termination of a stimulus, which is generally mediated by positive feedback loops (Pamenter & Powell, 2016). A glutamatergic pathway appears vital to the occurrence of acute hypoxic short‐term potentiation (Pamenter & Powell, 2016). The underlying mechanisms are uncertain, but it is possible that glutamate receptor activation increases the calcium concentration within postsynaptic NTS neurons, resulting in activation of calcium/calmodulin‐stimulated protein kinase II, which phosphorylates cytosolic neuronal nitric oxide synthase, stimulating production of NO (Kline et al., 2002; Pamenter & Powell, 2016). The NO then can diffuse back from the postsynaptic NTS neuron to the presynaptic afferent neuron, stimulating the production of cyclic guanosine monophosphate, enhancing glutamate release by the presynaptic afferent neuron (Kline et al., 2002; Pamenter & Powell, 2016). Whether NO in the NTS contributes to the exercise hyperpnoea remains unknown. However, greater NO availability in the NTS decreases cardiovascular responses to simulated exercise in rats (Leal et al., 2013, 2012). Then, if NO is involved in the exercise‐induced potentiation of the HVR, it might either act specifically in carotid chemoreceptor afferents but not in muscle afferent synapses with second‐order NTS neurons, or the NO effect might differ between NTS neurons projecting to respiratory and cardiovascular‐related neurons.

## 5 PERIPHERAL MECHANISMS UNDERLYING SYNERGISTIC INTERACTIONS BETWEEN NEURAL MECHANISMS

Increased sympathetic activity is a neural mechanism that might mediate the exercise‐induced HVR potentiation in the periphery (Figure 4). The carotid bodies receive sympathetic efferent innervation from the superior cervical ganglion (Biscoe & Purves, 1967; Bowers & Zigmond, 1979). Electrical activation of such efferent sympathetic fibres in anaesthetized cats (Floyd & Neil, 1952; Szulczyk & Trzebski, 1977) and in a rat preparation (Felippe et al., 2023) increases the carotid sinus nerve afferent activity. Sympathetic fibres innervate the blood vessels supplying the carotid bodies and can regulate the blood flow to the carotid bodies (O'Regan, 1981). In addition, via release of neurotransmitters into the interstitial space, sympathetic fibres can act directly on carotid body cells and carotid body afferents (i.e., petrosal ganglion afferents) (Ichikawa, 2002; McDonald & Mitchell, 1975). Sympathetic efferents to the carotid bodies secrete noradrenaline, neuropeptide Y, vasoactive intestinal peptide and acetylcholine (Brognara et al., 2021). These neurotransmitters can act on α_1_‐, α_2_‐, β_1_‐ and β_2_‐adrenoreceptors and dopamine 2, neuropeptide Y and vasoactive intestinal peptide receptors expressed on blood vessels, carotid body cells or carotid body afferents (Brognara et al., 2021). Therefore, the net effect of sympathetic activation on the activity of the carotid bodies depends, theoretically, on a complex interplay between excitatory and inhibitory effects mediated by the previously mentioned neurotransmitters acting on different sites (Zera et al., 2019).

The autonomic contribution to the resting HVR seems trivial in healthy humans (Liu et al., 2007), given that systemic infusion of trimethaphan to block the cholinergic transmission from pre‐ to postganglionic autonomic neurons does not change the acute HVR at sea level and high altitude. Some studies have investigated whether β‐receptor antagonists and agonists influence the ventilatory response during hypoxic exercise in goats (Pizarro et al., 1992; Warner & Mitchell, 1991; Weil et al., 1972) and humans (Agostoni et al., 2006; Conway & Petersen, 1987; Weil et al., 1972). However, the drugs were delivered systemically in resting and exercise studies, generating multiple effects beyond those on the carotid bodies. In addition, sympathetic nerves release neurotransmitters other than catecholamines that interact with various receptors. Superior cervical ganglion denervation in animals circumvents these limitations, because it eliminates the sympathetic efferents to the carotid bodies, without systemic effects. In healthy mice (Getsy et al., 2021), rats (Getsy et al., 2023) and goats (Ryan et al., 1995), superior cervical ganglion denervation does not change the resting HVR or provokes only minor effects. However, findings from a recent study conducted in spontaneously hypertensive rats showed that the same procedure decreases the HVR in the working brain–heart preparation via α_1_‐adrenoreceptors and lowers blood pressure in vivo in free‐living animals (Felippe et al., 2023). The same study found minor effects of superior cervical ganglion denervation in control Wistar rats. Results from another recent study showed that bilateral removal of the superior cervical ganglion does not change resting pulmonary ventilation or the HVR in healthy rats, but the HVR was enhanced after bleomycin‐induced acute lung injury, and this enhanced response was attenuated after removal of the superior cervical ganglia (Kamra et al., 2023). Collectively, recent data suggest that the influence of sympathetic efferents on carotid chemoreflex‐mediated responses is enhanced in animal models characterized by enhanced carotid chemoreceptor activity and sensitivity. However, whether the ventilatory response to hypoxic exercise depends on the sympathetic regulation of carotid bodies remains uncertain.

In addition to the carotid body effects,elevated sympathetic activity as a result of combined hypoxia and high‐intensity exercise could result in restraint of skeletal muscle blood flow in patients with chronic systolic heart failure (Di Vanna et al., 2007) and, maybe, in other patients with high sympathetic activity and poor vascular function, such as patients with chronic obstructive pulmonary disease (Ribeiro et al., 2021). The blood flow restraint might compromise O_2_ delivery to, and metabolite removal from, contracting muscles. Then, the restraint might result in a greater accumulation of metabolites in contracting muscles, thereby influencing excitation–contraction coupling and enhancing the activation of skeletal muscle metaboreceptors. In contrast, the greater sympathetic activity during hypoxic exercise in healthy young adults is counteracted by local compensatory vasodilatory mechanisms (Joyner & Casey, 2014) and is therefore unlikely to represent a limiting factor for matching blood flow to the metabolic demand of contracting skeletal muscles (Stickland et al., 2011).

## 6 CEREBRAL BLOOD FLOW

**FIGURE 5:**
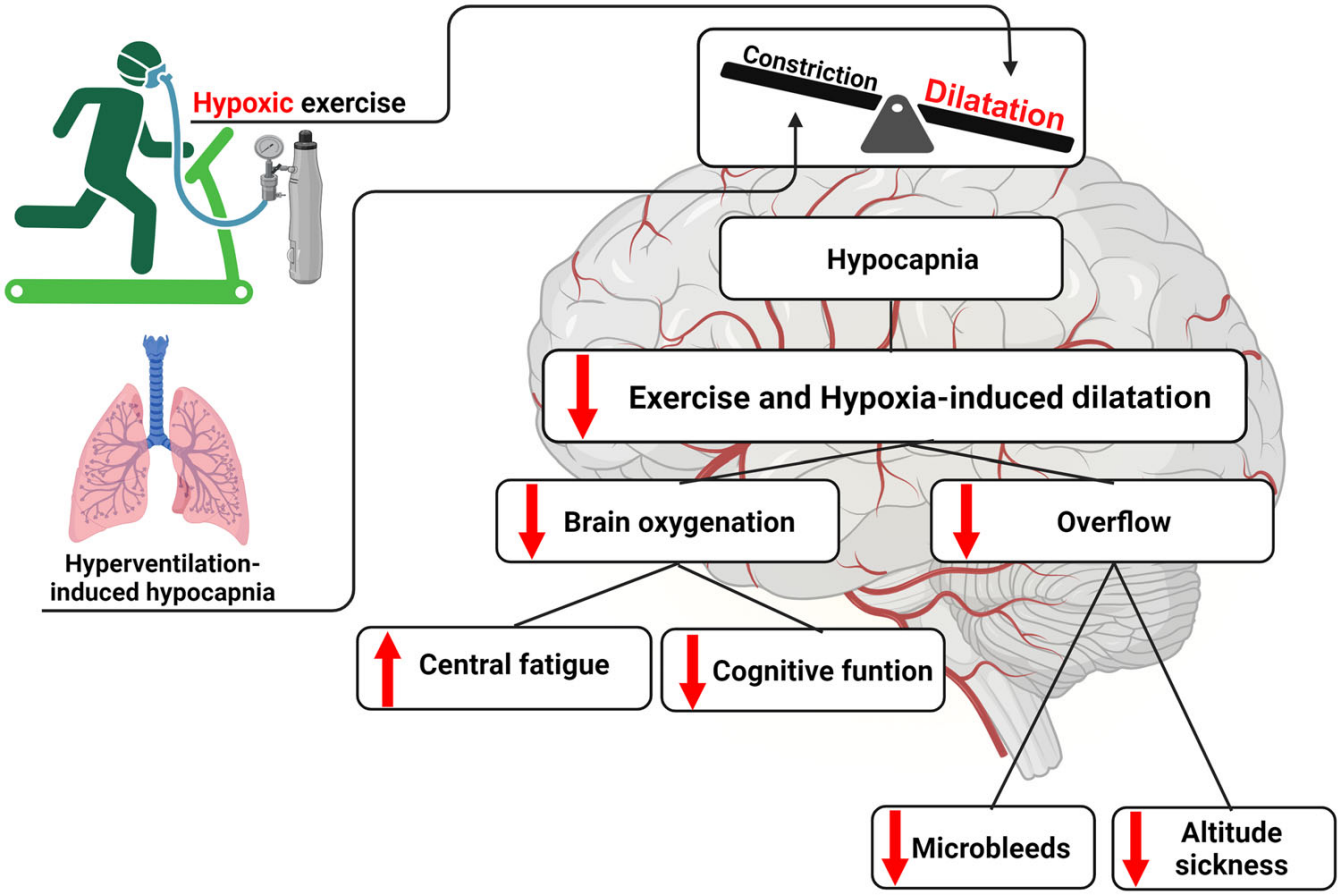
Potential consequences of hypoxic exercise on cerebrovascular regulation. Hypoxia and exercise generate dilatation, whereas hyperventilation‐induced hypocapnia generates constriction. Dilatation prevails. However, hypocapnia reduces exercise‐ and hypoxia‐induced dilatation, decreasing brain oxygenation and cerebral perfusion. The decreased oxygenation generates central fatigue, might impair cognitive performance and might decrease the possibility of microbleeds and occurrence of altitude sickness.

The hypoxia‐induced stimulation of the carotid chemoreflex elevates the ventilatory response to exercise, generating hypocapnia even during moderate (i.e., below the lactate threshold) and heavy‐intensity exercise (i.e., between the lactate threshold and the critical power) and accentuating the decrease in the arterial partial pressure of CO_2_ during severe‐intensity exercise (i.e., above critical power) (Doutreleau et al., 2017; Hansen et al., 2020; Siebenmann et al., 2013). The cerebrovascular response to hypoxic exercise ultimately depends on the interplay of the effects of hypoxia, neuronal activity and hypocapnia (Ogoh et al., 2014), because hypoxia and neuronal activity per se elicit vasodilatation, whereas hypocapnia elicits vasoconstriction (Bailey et al., 2018, 2017; Verges et al., 2012; Figure 5). Some studies have reported greater mean blood velocity (i.e., a surrogate of cerebral blood flow) in the middle cerebral artery (MCAv) during hypoxic exercise than during normoxic exercise performed at the same absolute or relative workload in healthy adults (Verges et al., 2012), indicating that vasodilator effects overcome vasoconstrictor effects.

Although cerebral vasodilatation prevails during hypoxic exercise, hypocapnia is not without effect, because experimental prevention of hypocapnia during hypoxic exercise supports the notion that hypocapnia restrains some of the vasodilator effects in some circumstances. For instance, CO_2_ supplementation in the inhaled air to the clamp arterial partial pressure of CO_2_ during hypoxic exercise increases prefrontal cortex oxygenation during small (i.e., single‐leg kicking; Rupp et al., 2015) and large muscle mass (i.e., cycling) dynamic exercise (Fan et al., 2013; Siebenmann et al., 2013; Subudhi et al., 2011) and increases MCAv during large muscle mass dynamic exercise (i.e., cycling) (Fan et al., 2013; Siebenmann et al., 2013; Subudhi et al., 2011), supporting the contention that hypocapnia counteracts the hypoxia‐induced cerebral vasodilatation. However, this effect might depend on acclimatization to high altitude, because resting cerebrovascular reactivity to CO_2_ increases after acclimatization to high altitude (Fan et al., 2010), which deserves investigation.

## 7 CLINICAL RELEVANCE

The hypocapnic restraint of hypoxia‐induced cerebral vasodilatation might have functional consequences. For instance, during small muscle mass exercise, avoidance of hypocapnia decreases central fatigue but, at the same time, exacerbates peripheral fatigue and does not change exercise performance (Rupp et al., 2015). The avoidance of hypocapnia improves cognitive function, as assessed by single reaction time and five‐choice reaction time, during exposure to hypoxia at rest (Friend et al., 2019). However, the effect of hypoxia on cognitive function is modified by exercise. Although exercise generally enhances cognition, severe hypoxia can counteract these benefits (Ando et al., 2020). Whether avoidance of hypocapnia enhances cognitive function during hypoxic exercise remains to be investigated, but it is a plausible hypothesis, because CO_2_ supplementation during hypoxic exercise restores cerebral O_2_ delivery to a normoxic exercise level during large muscle group dynamic exercise performed at submaximal and maximal exercise intensities (Fan et al., 2013; Siebenmann et al., 2013; Subudhi et al., 2011).

The restriction of hypoxia‐induced vasodilatation in the presence of hyperventilation‐induced hypocapnia during submaximal and maximal dynamic exercise could, hypothetically, avoid an excessive elevation in cerebral blood flow, thereby preventing fluid extravasation into the cerebral interstitium (Dubowitz et al., 2009) and microbleeds (Hackett et al., 2019). These hypocapnia‐related effects might be functionally relevant in the context of high‐altitude sickness, but they remain unproven and deserve investigation. In contrast, a hypocapnia‐related restriction of cerebral perfusion does not seem to hold during supramaximal exercise (i.e., sprint) in young healthy participants. Severe hypoxia during sprint exercise does not change cerebral O_2_ delivery in comparison to normoxic sprint exercise, despite a large increase in perfusion pressure and MCAv (Curtelin et al., 2018), suggesting that in these conditions, vasodilator effects abolish vasoconstrictor effects in favour of the maintenance of O_2_ delivery despite the potential increase in cerebral haemodynamic risk (Calverley et al., 2020). However, this notion requires further scrutiny, given that no study has tested the effect of CO_2_ clamping during hypoxic sprint exercise.

It is noteworthy that some diseases exacerbate the ventilatory response to exercise. Some examples include chronic heart failure (Guazzi et al., 2005) and pulmonary arterial hypertension (Malenfant et al., 2020, 2017; Sayegh et al., 2021). The heightened hyperventilation‐induced hypocapnia in these patients is clinically significant, correlating with the severity of exercise intolerance and increased risk of mortality (Guazzi et al., 2005; Sayegh et al., 2021). Additionally, these patients exhibit diminished cerebrovascular reactivity to CO_2_ alongside an exaggerated ventilatory response (Georgiadis et al., 2000; Malenfant et al., 2017). Whether an exaggerated hypocapnia during exercise provokes restriction of the increase in cerebral blood flow in these patients remains to be clearly determined (Brassard & Gustafsson, 2016). If the increase in cerebral blood flow is compromised by hypocapnia, it is plausible that hypocapnia might accentuate the appearance of central fatigue and impair cognitive performance.

## 8 CONCLUSIONS

The intricate interplay of neural mechanisms orchestrates the exercise‐induced potentiation of the HVR. This phenomenon operates independently of humoral factors, showcasing the remarkable capacity of neural pathways to mediate the HVR during physical exertion. Although current evidence discounts a direct interaction between the carotid chemoreflex and central command, we present evidence of synergies between the carotid chemoreflex with muscle mechano‐ and metaboreflexes. Spatial and temporal summation of afferent signals, short‐term potentiation and sympathetically induced carotid chemoreceptor activation potentially mediate the exercise‐induced HVR potentiation. Furthermore, the amplified HVR during exercise induces hyperventilation‐driven hypocapnia, impacting cerebral blood flow regulation, with diverse potential consequences, including heightened central fatigue, impaired cognitive function and safeguarding against excessive cerebral perfusion.

## AUTHOR CONTRIBUTIONS

Diogo Machado de Oliveira: Conception or design of the work and drafting of the work or revising it critically for important intellectual content. Anas Rashid: Conception or design of the work and drafting of the work or revising it critically for important intellectual content. Patrice Brassard: Conception or design of the work and drafting of the work or revising it critically for important intellectual content. Bruno Moreira Silva: Conception or design of the work and drafting of the work or revising it critically for important intellectual content. All authors have read and approved the final version of this manuscript and agree to be accountable for all aspects of the work in ensuring that questions related to the accuracy or integrity of any part of the work are appropriately investigated and resolved. All persons designated as authors qualify for authorship, and all those who qualify for authorship are listed.

## CONFLICT OF INTEREST

The authors declare no conflicts of interest.

## References

[eph13506-bib-0001] Agostoni, P. , Contini, M. , Magini, A. , Apostolo, A. , Cattadori, G. , Bussotti, M. , Veglia, F. , Andreini, D. , & Palermo, P. (2006). Carvedilol reduces exercise‐induced hyperventilation: A benefit in normoxia and a problem with hypoxia. European Journal of Heart Failure, 8(7), 729–735. 16533619 10.1016/j.ejheart.2006.02.001

[eph13506-bib-0002] Ando, S. , Komiyama, T. , Sudo, M. , Higaki, Y. , Ishida, K. , Costello, J. T. , & Katayama, K. (2020). The interactive effects of acute exercise and hypoxia on cognitive performance: A narrative review. Scandinavian Journal of Medicine & Science in Sports, 30(3), 384–398. 31605635 10.1111/sms.13573

[eph13506-bib-0003] Aranda, L. C. , Ribeiro, I. C. , Freitas, T. O. , Degani‐Costa, L. H. , Dias, D. S. , De Angelis, K. , Paixão, A. O. , Brum, P. C. , Oliveira, A. S. B. , Vianna, L. C. , Nery, L. E. , & Silva, B. M. (2024). Altered locomotor muscle metaboreflex control of ventilation in patients with COPD. Journal of Applied Physiology (1985), 136(2), 385–398. 10.1152/japplphysiol.00560.2023 38174374

[eph13506-bib-0004] Bailey, D. M. , Rasmussen, P. , Evans, K. A. , Bohm, A. M. , Zaar, M. , Nielsen, H. B. , Brassard, P. , Nordsborg, N. B. , Homann, P. H. , Raven, P. B. , McEneny, J. , Young, I. S. , McCord, J. M. , & Secher, N. H. (2018). Hypoxia compounds exercise‐induced free radical formation in humans; partitioning contributions from the cerebral and femoral circulation. Free Radical Biology and Medicine, 124, 104–113. 29859345 10.1016/j.freeradbiomed.2018.05.090

[eph13506-bib-0005] Bailey, D. M. , Rasmussen, P. , Overgaard, M. , Evans, K. A. , Bohm, A. M. , Seifert, T. , Brassard, P. , Zaar, M. , Nielsen, H. B. , Raven, P. B. , & Secher, N. H. (2017). Nitrite and S‐nitrosohemoglobin exchange across the human cerebral and femoral circulation: Relationship to basal and exercise blood flow responses to hypoxia. Circulation, 135(2), 166–176. 27881556 10.1161/CIRCULATIONAHA.116.024226

[eph13506-bib-0006] Barna, B. F. , Takakura, A. C. , & Moreira, T. S. (2012). Pontomedullary and hypothalamic distribution of Fos‐like immunoreactive neurons after acute exercise in rats. Neuroscience, 212, 120–130. 22521827 10.1016/j.neuroscience.2012.03.039

[eph13506-bib-0007] Barna, B. F. , Takakura, A. C. , & Moreira, T. S. (2014). Acute exercise‐induced activation of Phox2b‐expressing neurons of the retrotrapezoid nucleus in rats may involve the hypothalamus. Neuroscience, 258, 355–363. 24286756 10.1016/j.neuroscience.2013.11.031

[eph13506-bib-0008] Bendahan, D. , Jammes, Y. , Salvan, A. M. , Badier, M. , Confort‐Gouny, S. , Guillot, C. , & Cozzone, P. J. (1996). Combined electromyography‐31P‐magnetic resonance spectroscopy study of human muscle fatigue during static contraction. Muscle & Nerve, 19(6), 715–721. 8609921 10.1002/(SICI)1097-4598(199606)19:6<715::AID-MUS5>3.0.CO;2-D

[eph13506-bib-0009] Biscoe, T. J. , & Purves, M. J. (1967). Observations on carotid body chemoreceptor activity and cervical sympathetic discharge in the cat. The Journal of Physiology, 190(3), 413–424. 6051779 10.1113/jphysiol.1967.sp008218 PMC1365418

[eph13506-bib-0010] Bowers, C. W. , & Zigmond, R. E. (1979). Localization of neurons in the rat superior cervical ganglion that project into different postganglionic trunks. Journal of Comparative Neurology, 185(2), 381–391. 429622 10.1002/cne.901850211

[eph13506-bib-0011] Brassard, P. , & Gustafsson, F. (2016). Exercise intolerance in heart failure: Did we forget the brain? Canadian Journal of Cardiology, 32(4), 475–484. 26875016 10.1016/j.cjca.2015.12.021

[eph13506-bib-0012] Brognara, F. , Felippe, I. S. A. , Salgado, H. C. , & Paton, J. F. R. (2021). Autonomic innervation of the carotid body as a determinant of its sensitivity: Implications for cardiovascular physiology and pathology. Cardiovascular Research, 117(4), 1015–1032. 32832979 10.1093/cvr/cvaa250

[eph13506-bib-0013] Bruce, R. M. , & White, M. J. (2012). Muscle afferent activation causes ventilatory and cardiovascular responses during concurrent hypercapnia in humans. Experimental Physiology, 97(2), 208–218. 22058167 10.1113/expphysiol.2011.061606

[eph13506-bib-0014] Calverley, T. A. , Ogoh, S. , Marley, C. J. , Steggall, M. , Marchi, N. , Brassard, P. , Lucas, S. J. E. , Cotter, J. D. , Roig, M. , Ainslie, P. N. , Wisløff, U. , & Bailey, D. M. (2020). HIITing the brain with exercise: Mechanisms, consequences and practical recommendations. The Journal of Physiology, 598(13), 2513–2530. 32347544 10.1113/JP275021

[eph13506-bib-0015] Conway, M. A. , & Petersen, E. S. (1987). Effects of beta‐adrenergic blockade on the ventilatory responses to hypoxic and hyperoxic exercise in man. The Journal of Physiology, 393, 43–55. 3446803 10.1113/jphysiol.1987.sp016809 PMC1192379

[eph13506-bib-0016] Curtelin, D. , Morales‐Alamo, D. , Torres‐Peralta, R. , Rasmussen, P. , Martin‐Rincon, M. , Perez‐Valera, M. , Siebenmann, C. , Pérez‐Suárez, I. , Cherouveim, E. , Sheel, A. W. , Lundby, C. , & Calbet, J. A. (2018). Cerebral blood flow, frontal lobe oxygenation and intra‐arterial blood pressure during sprint exercise in normoxia and severe acute hypoxia in humans. Journal of Cerebral Blood Flow and Metabolism, 38(1), 136–150. 28186430 10.1177/0271678X17691986 PMC5757439

[eph13506-bib-0017] Cutsforth‐Gregory, J. K. , & Benarroch, E. E. (2017). Nucleus of the solitary tract, medullary reflexes, and clinical implications. Neurology, 88(12), 1187–1196. 28202704 10.1212/WNL.0000000000003751

[eph13506-bib-0018] de Oliveira, D. M. , Lopes, T. R. , Gomes, F. S. , Rashid, A. , & Silva, B. M. (2023). Ventilatory response to peripheral chemoreflex and muscle metaboreflex during static handgrip in healthy humans: Evidence of hyperadditive integration. Experimental Physiology, 108(7), 932–939. 37036125 10.1113/EP091094 PMC10988439

[eph13506-bib-0019] Di Vanna, A. , Braga, A. M. , Laterza, M. C. , Ueno, L. M. , Rondon, M. U. , Barretto, A. C. , Middlekauff, H. R. , & Negrão, C. E. (2007). Blunted muscle vasodilatation during chemoreceptor stimulation in patients with heart failure. American Journal of Physiology‐Heart and Circulatory Physiology, 293(1), H846–852. 17434973 10.1152/ajpheart.00156.2007

[eph13506-bib-0020] Doutreleau, S. , Enache, I. , Pistea, C. , Favret, F. , Lonsdorfer, E. , Dufour, S. , & Charloux, A. (2017). Cardio‐respiratory responses to hypoxia combined with CO(2) inhalation during maximal exercise. Respiratory Physiology & Neurobiology, 235, 52–61. 27688122 10.1016/j.resp.2016.09.012

[eph13506-bib-0021] Dubowitz, D. J. , Dyer, E. A. , Theilmann, R. J. , Buxton, R. B. , & Hopkins, S. R. (2009). Early brain swelling in acute hypoxia. Journal of Applied Physiology, 107(1), 244–252. 19423837 10.1152/japplphysiol.90349.2008 PMC2711789

[eph13506-bib-0022] Edgell, H. , & Stickland, M. K. (2014). Activation of the carotid chemoreflex secondary to muscle metaboreflex stimulation in men. American Journal of Physiology‐Regulatory, Integrative and Comparative Physiology, 306(9), R693–R700. 24573180 10.1152/ajpregu.00472.2013

[eph13506-bib-0023] Fan, J. L. , Bourdillon, N. , & Kayser, B. (2013). Effect of end‐tidal CO2 clamping on cerebrovascular function, oxygenation, and performance during 15‐km time trial cycling in severe normobaric hypoxia: The role of cerebral O2 delivery. Physiological Reports, 1(3), e00066. 24303142 10.1002/phy2.66 PMC3835019

[eph13506-bib-0024] Fan, J. L. , Burgess, K. R. , Basnyat, R. , Thomas, K. N. , Peebles, K. C. , Lucas, S. J. , Lucas, R. A. , Donnelly, J. , Cotter, J. D. , & Ainslie, P. N. (2010). Influence of high altitude on cerebrovascular and ventilatory responsiveness to CO2. The Journal of Physiology, 588(Pt 3), 539–549. 20026618 10.1113/jphysiol.2009.184051 PMC2825616

[eph13506-bib-0025] Felippe, I. S. A. , Zera, T. , da Silva, M. P. , Moraes, D. J. A. , McBryde, F. , & Paton, J. F. R. (2023). The sympathetic nervous system exacerbates carotid body sensitivity in hypertension. Cardiovascular Research, 119(1), 316–331. 35048948 10.1093/cvr/cvac008 PMC10022867

[eph13506-bib-0026] Floyd, W. F. , & Neil, E. (1952). The influence of the sympathetic innervation of the carotid bifurcation on chemoceptor and baroceptor activity in the cat. Archives Internationales De Pharmacodynamie Et De Therapie, 91(1‐2), 230–239. 13008503

[eph13506-bib-0027] Fregosi, R. F. , & Seals, D. R. (1993). Hypoxic potentiation of the ventilatory response to dynamic forearm exercise. Journal of Applied Physiology, 74(5), 2365–2372. 8335569 10.1152/jappl.1993.74.5.2365

[eph13506-bib-0028] Friend, A. T. , Balanos, G. M. , & Lucas, S. J. E. (2019). Isolating the independent effects of hypoxia and hyperventilation‐induced hypocapnia on cerebral haemodynamics and cognitive function. Experimental Physiology, 104(10), 1482–1493. 31342596 10.1113/EP087602

[eph13506-bib-0029] Fukuoka, Y. , Endo, M. , Oishi, Y. , & Ikegami, H. (2003). Chemoreflex drive and the dynamics of ventilation and gas exchange during exercise at hypoxia. American Journal of Respiratory and Critical Care Medicine, 168(9), 1115–1122. 14581289 10.1164/rccm.2202027

[eph13506-bib-0030] Georgiadis, D. , Sievert, M. , Cencetti, S. , Uhlmann, F. , Krivokuca, M. , Zierz, S. , & Werdan, K. (2000). Cerebrovascular reactivity is impaired in patients with cardiac failure. European Heart Journal, 21(5), 407–413. 10666355 10.1053/euhj.1999.1742

[eph13506-bib-0031] Getsy, P. M. , Coffee, G. A. , Hsieh, Y. H. , & Lewis, S. J. (2021). The superior cervical ganglia modulate ventilatory responses to hypoxia independently of preganglionic drive from the cervical sympathetic chain. Journal of Applied Physiology, 131(2), 836–857. 34197230 10.1152/japplphysiol.00216.2021 PMC8409919

[eph13506-bib-0032] Getsy, P. M. , Coffee, G. A. , & Lewis, S. J. (2023). Loss of ganglioglomerular nerve input to the carotid body impacts the hypoxic ventilatory response in freely‐moving rats. Frontiers in Physiology, 14, 1007043. 37008015 10.3389/fphys.2023.1007043 PMC10060956

[eph13506-bib-0033] Guazzi, M. , Reina, G. , Tumminello, G. , & Guazzi, M. D. (2005). Exercise ventilation inefficiency and cardiovascular mortality in heart failure: The critical independent prognostic value of the arterial CO2 partial pressure. European Heart Journal, 26(5), 472–480. 15618042 10.1093/eurheartj/ehi060

[eph13506-bib-0034] Gujic, M. , Laude, D. , Houssière, A. , Beloka, S. , Argacha, J. F. , Adamopoulos, D. , Xhaët, O. , Elghozi, J. L. , & van de Borne, P. (2007). Differential effects of metaboreceptor and chemoreceptor activation on sympathetic and cardiac baroreflex control following exercise in hypoxia in human. The Journal of Physiology, 585(Pt 1), 165–174. 17884922 10.1113/jphysiol.2007.141002 PMC2375466

[eph13506-bib-0035] Hackett, P. H. , Yarnell, P. R. , Weiland, D. A. , & Reynard, K. B. (2019). Acute and evolving MRI of high‐altitude cerebral edema: Microbleeds, edema, and pathophysiology. AJNR American Journal of Neuroradiology, 40(3), 464–469. 30679208 10.3174/ajnr.A5897 PMC7028681

[eph13506-bib-0036] Hansen, R. K. , Nielsen, P. S. , Schelske, M. W. , Secher, N. H. , & Volianitis, S. (2020). CO(2) supplementation dissociates cerebral oxygenation and middle cerebral artery blood velocity during maximal cycling. Scandinavian Journal of Medicine & Science in Sports, 30(3), 399–407. 31650627 10.1111/sms.13582

[eph13506-bib-0037] Hargens, T. A. , Guill, S. G. , Aron, A. , Zedalis, D. , Gregg, J. M. , Nickols‐Richardson, S. M. , & Herbert, W. G. (2009). Altered ventilatory responses to exercise testing in young adult men with obstructive sleep apnea. Respiratory Medicine, 103(7), 1063–1069. 19217270 10.1016/j.rmed.2009.01.010

[eph13506-bib-0038] Hérent, C. , Diem, S. , Usseglio, G. , Fortin, G. , & Bouvier, J. (2023). Upregulation of breathing rate during running exercise by central locomotor circuits in mice. Nature Communications, 14(1), 2939. 10.1038/s41467-023-38583-6 PMC10203288 37217517

[eph13506-bib-0039] Houssiere, A. , Najem, B. , Ciarka, A. , Velez‐Roa, S. , Naeije, R. , & van de Borne, P. (2005). Chemoreflex and metaboreflex control during static hypoxic exercise. American Journal of Physiology‐Heart and Circulatory Physiology, 288(4), H1724–H1729. 15604123 10.1152/ajpheart.01043.2004

[eph13506-bib-0040] Ichikawa, H. (2002). Innervation of the carotid body: Immunohistochemical, denervation, and retrograde tracing studies. Microscopy Research and Technique, 59(3), 188–195. 12384963 10.1002/jemt.10193

[eph13506-bib-0041] Joyner, M. J. , & Casey, D. P. (2014). Muscle blood flow, hypoxia, and hypoperfusion. Journal of Applied Physiology, 116(7), 852–857. 23887898 10.1152/japplphysiol.00620.2013 PMC3972742

[eph13506-bib-0042] Kamra, K. , Karpuk, N. , Zucker, I. H. , Schultz, H. D. , & Wang, H. J. (2023). The superior cervical ganglion is involved in chronic chemoreflex sensitization during recovery from acute lung injury. Frontiers in Physiology, 14, 1101408. 36846321 10.3389/fphys.2023.1101408 PMC9944401

[eph13506-bib-0043] Kanbar, R. , Stornetta, R. L. , & Guyenet, P. G. (2016). Sciatic nerve stimulation activates the retrotrapezoid nucleus in anesthetized rats. Journal of Neurophysiology, 116(5), 2081–2092. 27512023 10.1152/jn.00543.2016 PMC5102315

[eph13506-bib-0044] Kaufman, M. P. , Longhurst, J. C. , Rybicki, K. J. , Wallach, J. H. , & Mitchell, J. H. (1983). Effects of static muscular contraction on impulse activity of groups III and IV afferents in cats. Journal of Applied Physiology: Respiratory, Environmental and Exercise Physiology, 55(1 Pt 1), 105–112. 6309712 10.1152/jappl.1983.55.1.105

[eph13506-bib-0045] Kline, D. D. , Overholt, J. L. , & Prabhakar, N. R. (2002). Mutant mice deficient in NOS‐1 exhibit attenuated long‐term facilitation and short‐term potentiation in breathing. The Journal of Physiology, 539(Pt 1), 309–315. 11850522 10.1113/jphysiol.2001.014571 PMC2290125

[eph13506-bib-0046] Korsak, A. , Sheikhbahaei, S. , Machhada, A. , Gourine, A. V. , & Huckstepp, R. T. R. (2018). The role of parafacial neurons in the control of breathing during exercise. Scientific Reports, 8(1), 400. 29321559 10.1038/s41598-017-17412-z PMC5762684

[eph13506-bib-0047] Lam, E. , Greenhough, E. , Nazari, P. , White, M. J. , & Bruce, R. M. (2019). Muscle metaboreflex activation increases ventilation and heart rate during dynamic exercise in humans. Experimental Physiology, 104(10), 1472–1481. 31206823 10.1113/EP087726

[eph13506-bib-0048] Leal, A. K. , Mitchell, J. H. , & Smith, S. A. (2013). Treatment of muscle mechanoreflex dysfunction in hypertension: Effects of L‐arginine dialysis in the nucleus tractus solitarii. Experimental Physiology, 98(9), 1337–1348. 23771911 10.1113/expphysiol.2012.071563 PMC3750060

[eph13506-bib-0049] Leal, A. K. , Murphy, M. N. , Iwamoto, G. A. , Mitchell, J. H. , & Smith, S. A. (2012). A role for nitric oxide within the nucleus tractus solitarii in the development of muscle mechanoreflex dysfunction in hypertension. Experimental Physiology, 97(12), 1292–1304. 22581746 10.1113/expphysiol.2012.065433 PMC3480555

[eph13506-bib-0050] Li, Y. L. , Xia, X. H. , Zheng, H. , Gao, L. , Li, Y. F. , Liu, D. , Patel, K. P. , Wang, W. , & Schultz, H. D. (2006). Angiotensin II enhances carotid body chemoreflex control of sympathetic outflow in chronic heart failure rabbits. Cardiovascular Research, 71(1), 129–138. 16650840 10.1016/j.cardiores.2006.03.017

[eph13506-bib-0051] Lindefors, N. , Yamamoto, Y. , Pantaleo, T. , Lagercrantz, H. , Brodin, E. , & Ungerstedt, U. (1986). In vivo release of substance P in the nucleus tractus solitarii increases during hypoxia. Neuroscience Letters, 69(1), 94–97. 2427979 10.1016/0304-3940(86)90421-0

[eph13506-bib-0052] Liu, C. , Smith, T. G. , Balanos, G. M. , Brooks, J. , Crosby, A. , Herigstad, M. , Dorrington, K. L. , & Robbins, P. A. (2007). Lack of involvement of the autonomic nervous system in early ventilatory and pulmonary vascular acclimatization to hypoxia in humans. The Journal of Physiology, 579(Pt 1), 215–225. 17138611 10.1113/jphysiol.2006.118190 PMC1865001

[eph13506-bib-0053] Machado, A. C. , Vianna, L. C. , Gomes, E. A. C. , Teixeira, J. A. C. , Ribeiro, M. L. , Villacorta, H. , Nobrega, A. C. L. , & Silva, B. M. (2020). Carotid chemoreflex and muscle metaboreflex interact to the regulation of ventilation in patients with heart failure with reduced ejection fraction. Physiological Reports, 8(3), e14361. 32026605 10.14814/phy2.14361 PMC7002537

[eph13506-bib-0054] Malenfant, S. , Brassard, P. , Paquette, M. , Le Blanc, O. , Chouinard, A. , Bonnet, S. , & Provencher, S. (2020). Continuous reduction in cerebral oxygenation during endurance exercise in patients with pulmonary arterial hypertension. Physiological Reports, 8(6), e14389. 32189447 10.14814/phy2.14389 PMC7080869

[eph13506-bib-0055] Malenfant, S. , Brassard, P. , Paquette, M. , Le Blanc, O. , Chouinard, A. , Nadeau, V. , Allan, P. D. , Tzeng, Y. C. , Simard, S. , Bonnet, S. , & Provencher, S. (2017). Compromised cerebrovascular regulation and cerebral oxygenation in pulmonary arterial hypertension. Journal of the American Heart Association, 6(10), e006126. 29025748 10.1161/JAHA.117.006126 PMC5721836

[eph13506-bib-0056] McDonald, D. M. , & Mitchell, R. A. (1975). The innervation of glomus cells, ganglion cells and blood vessels in the rat carotid body: A quantitative ultrastructural analysis. Journal of Neurocytology, 4, 177–230.

[eph13506-bib-0057] Narkiewicz, K. , van de Borne, P. J. , Pesek, C. A. , Dyken, M. E. , Montano, N. , & Somers, V. K. (1999). Selective potentiation of peripheral chemoreflex sensitivity in obstructive sleep apnea. Circulation, 99(9), 1183–1189. 10069786 10.1161/01.cir.99.9.1183

[eph13506-bib-0058] O'Regan, R. G. (1981). Responses of carotid body chemosensory activity and blood flow to stimulation of sympathetic nerves in the cat. The Journal of Physiology, 315, 81–98. 7310725 10.1113/jphysiol.1981.sp013734 PMC1249369

[eph13506-bib-0059] Ogoh, S. , Nakahara, H. , Ueda, S. , Okazaki, K. , Shibasaki, M. , Subudhi, A. W. , & Miyamoto, T. (2014). Effects of acute hypoxia on cerebrovascular responses to carbon dioxide. Experimental Physiology, 99(6), 849–858. 24632495 10.1113/expphysiol.2013.076802

[eph13506-bib-0060] Pamenter, M. E. , & Powell, F. L. (2016). Time domains of the hypoxic ventilatory response and their molecular basis. Comprehensive Physiology, 6(3), 1345–1385. 27347896 10.1002/cphy.c150026 PMC4934681

[eph13506-bib-0061] Pandit, J. J. , & Robbins, P. A. (1994). Acute ventilatory responses to hypoxia during voluntary and electrically induced leg exercise in man. The Journal of Physiology, 477(Pt 1), 161–168. 8071883 10.1113/jphysiol.1994.sp020180 PMC1155583

[eph13506-bib-0062] Paula‐Ribeiro, M. , Ribeiro, I. C. , Aranda, L. C. , Silva, T. M. , Costa, C. M. , Ramos, R. P. , Ota‐Arakaki, J. , Cravo, S. L. , Nery, L. E. , Stickland, M. K. , & Silva, B. M. (2021). Cardiac baroreflex dysfunction in patients with pulmonary arterial hypertension at rest and during orthostatic stress: Role of the peripheral chemoreflex. Journal of Applied Physiology, 131(2), 794–807. 34197227 10.1152/japplphysiol.00152.2021

[eph13506-bib-0063] Paula‐Ribeiro, M. , Ribeiro, I. C. , Aranda, L. C. , Silva, T. M. , Costa, C. M. , Ramos, R. P. , Ota‐Arakaki, J. S. , Cravo, S. L. , Nery, L. E. , Stickland, M. K. , & Silva, B. M. (2019). Carotid chemoreflex activity restrains post‐exercise cardiac autonomic control in healthy humans and in patients with pulmonary arterial hypertension. The Journal of Physiology, 597(5), 1347–1360. 30628073 10.1113/JP277190 PMC6395424

[eph13506-bib-0064] Pizarro, J. , Warner, M. M. , Ryan, M. , Mitchell, G. S. , & Bisgard, G. E. (1992). Intracarotid norepinephrine infusions inhibit ventilation in goats. Respiration Physiology, 90(3), 299–310. 1480841 10.1016/0034-5687(92)90110-i

[eph13506-bib-0065] Plunkett, M. J. , Sayegh, A. L. C. , McWilliams, T. J. , Sithamparanathan, S. , Paton, J. F. R. , & Fisher, J. P. (2024). The skeletal muscle metaboreflex: A novel driver of ventilation, dyspnoea and pulmonary haemodynamics during exercise in pulmonary arterial hypertension. European Respiratory Journal, 63(1), 2300952. 37678950 10.1183/13993003.00952-2023 PMC10764981

[eph13506-bib-0066] Potts, J. T. , Fong, A. Y. , Anguelov, P. I. , Lee, S. , McGovern, D. , & Grias, I. (2007). Targeted deletion of neurokinin‐1 receptor expressing nucleus tractus solitarii neurons precludes somatosensory depression of arterial baroreceptor‐heart rate reflex. Neuroscience, 145(3), 1168–1181. 17293052 10.1016/j.neuroscience.2007.01.001 PMC1905828

[eph13506-bib-0067] Potts, J. T. , Fuchs, I. E. , Li, J. , Leshnower, B. , & Mitchell, J. H. (1999). Skeletal muscle afferent fibres release substance P in the nucleus tractus solitarii of anaesthetized cats. The Journal of Physiology, 514(Pt 3), 829–841. 9882754 10.1111/j.1469-7793.1999.829ad.x PMC2269110

[eph13506-bib-0068] Potts, J. T. , & Waldrop, T. G. (2005). Discharge patterns of somatosensitive neurons in the nucleus tractus solitarius of the cat. Neuroscience, 132(4), 1123–1134. 15857716 10.1016/j.neuroscience.2004.12.018

[eph13506-bib-0069] Regensteiner, J. G. , Pickett, C. K. , McCullough, R. E. , Weil, J. V. , & Moore, L. G. (1988). Possible gender differences in the effect of exercise on hypoxic ventilatory response. Respiration, 53(3), 158–165. 3138747 10.1159/000195409

[eph13506-bib-0070] Ribeiro, I. C. , Aranda, L. C. , Freitas, T. O. , Degani‐Costa, L. H. , Ferreira, E. V. M. , Nery, L. E. , & Silva, B. M. (2021). Intercostal and vastus lateralis microcirculatory response to a sympathoexcitatory manoeuvre in patients with chronic obstructive pulmonary disease. Respiratory Physiology & Neurobiology, 290, 103678. 33957298 10.1016/j.resp.2021.103678

[eph13506-bib-0071] Rosin, D. L. , Chang, D. A. , & Guyenet, P. G. (2006). Afferent and efferent connections of the rat retrotrapezoid nucleus. Journal of Comparative Neurology, 499(1), 64–89. 16958085 10.1002/cne.21105

[eph13506-bib-0072] Rupp, T. , Mallouf Tle, R. , Perrey, S. , Wuyam, B. , Millet, G. Y. , & Verges, S. (2015). CO2 clamping, peripheral and central fatigue during hypoxic knee extensions in men. Medicine and Science in Sports and Exercise, 47(12), 2513–2524. 26110698 10.1249/MSS.0000000000000724

[eph13506-bib-0073] Ryan, M. L. , Hedrick, M. S. , Pizarro, J. , & Bisgard, G. E. (1995). Effects of carotid body sympathetic denervation on ventilatory acclimatization to hypoxia in the goat. Respiration Physiology, 99(2), 215–224. 7777704 10.1016/0034-5687(94)00096-i

[eph13506-bib-0074] Sayegh, A. L. C. , Silva, B. M. , Ferreira, E. V. M. , Ramos, R. P. , Fisher, J. P. , Nery, L. E. , Ota‐Arakaki, J. S. , & Oliveira, R. K. F. (2021). Clinical utility of ventilatory and gas exchange evaluation during low‐intensity exercise for risk stratification and prognostication in pulmonary arterial hypertension. Respirology (Carlton, Vic.), 26(3), 264–272. 33118293 10.1111/resp.13959

[eph13506-bib-0075] Siebenmann, C. , Sørensen, H. , Jacobs, R. A. , Haider, T. , Rasmussen, P. , & Lundby, C. (2013). Hypocapnia during hypoxic exercise and its impact on cerebral oxygenation, ventilation and maximal whole body O₂ uptake. Respiratory Physiology & Neurobiology, 185(2), 461–467. 22922610 10.1016/j.resp.2012.08.012

[eph13506-bib-0076] Silva, T. M. , Aranda, L. C. , Paula‐Ribeiro, M. , Oliveira, D. M. , Medeiros, W. M. , Vianna, L. C. , Nery, L. E. , & Silva, B. M. (2018). Hyperadditive ventilatory response arising from interaction between the carotid chemoreflex and the muscle mechanoreflex in healthy humans. Journal of Applied Physiology, 125(1), 215–225. 29565769 10.1152/japplphysiol.00009.2018

[eph13506-bib-0077] Smith, J. C. , Greer, J. J. , Liu, G. S. , & Feldman, J. L. (1990). Neural mechanisms generating respiratory pattern in mammalian brain stem‐spinal cord in vitro. I. Spatiotemporal patterns of motor and medullary neuron activity. Journal of Neurophysiology, 64(4), 1149–1169. 2258739 10.1152/jn.1990.64.4.1149

[eph13506-bib-0078] Stickland, M. K. , Fuhr, D. P. , Edgell, H. , Byers, B. W. , Bhutani, M. , Wong, E. Y. , & Steinback, C. D. (2016). Chemosensitivity, cardiovascular risk, and the ventilatory response to exercise in COPD. PLoS ONE, 11(6), e0158341. 27355356 10.1371/journal.pone.0158341 PMC4927073

[eph13506-bib-0079] Stickland, M. K. , Fuhr, D. P. , Haykowsky, M. J. , Jones, K. E. , Paterson, D. I. , Ezekowitz, J. A. , & McMurtry, M. S. (2011). Carotid chemoreceptor modulation of blood flow during exercise in healthy humans. The Journal of Physiology, 589(Pt 24), 6219–6230. 22025661 10.1113/jphysiol.2011.218099 PMC3286697

[eph13506-bib-0080] Subudhi, A. W. , Olin, J. T. , Dimmen, A. C. , Polaner, D. M. , Kayser, B. , & Roach, R. C. (2011). Does cerebral oxygen delivery limit incremental exercise performance? Journal of Applied Physiology, 111(6), 1727–1734. 21921244 10.1152/japplphysiol.00569.2011 PMC3233884

[eph13506-bib-0081] Szulczyk, P. , & Trzebski, A. (1977). Effects of carotid chemoreceptor and baroreceptor stimulation upon the sympathetic preganglionic and postganglionic cardiac nerve and single fiber activity in cats. Acta Neurobiologiae Experimentalis (Wars), 31(1), 15–25. 855684

[eph13506-bib-0082] Takakura, A. C. , Moreira, T. S. , Colombari, E. , West, G. H. , Stornetta, R. L. , & Guyenet, P. G. (2006). Peripheral chemoreceptor inputs to retrotrapezoid nucleus (RTN) CO2‐sensitive neurons in rats. The Journal of Physiology, 572(Pt 2), 503–523. 16455687 10.1113/jphysiol.2005.103788 PMC1779666

[eph13506-bib-0083] Teppema, L. J. , Veening, J. G. , Kranenburg, A. , Dahan, A. , Berkenbosch, A. , & Olievier, C. (1997). Expression of c‐fos in the rat brainstem after exposure to hypoxia and to normoxic and hyperoxic hypercapnia. Journal of Comparative Neurology, 388(2), 169–190. 9368836 10.1002/(sici)1096-9861(19971117)388:2<169::aid-cne1>3.0.co;2-#

[eph13506-bib-0084] Timmers, H. J. , Wieling, W. , Karemaker, J. M. , & Lenders, J. W. (2003). Denervation of carotid baro‐ and chemoreceptors in humans. The Journal of Physiology, 553(Pt 1), 3–11. 14528027 10.1113/jphysiol.2003.052415 PMC2343492

[eph13506-bib-0085] Verges, S. , Rupp, T. , Jubeau, M. , Wuyam, B. , Esteve, F. , Levy, P. , Perrey, S. , & Millet, G. Y. (2012). Cerebral perturbations during exercise in hypoxia. American Journal of Physiology‐Regulatory, Integrative and Comparative Physiology, 302(8), R903–R916. 22319046 10.1152/ajpregu.00555.2011

[eph13506-bib-0086] Wan, H. Y. , Weavil, J. C. , Thurston, T. S. , Georgescu, V. P. , Bledsoe, A. D. , Jessop, J. E. , Buys, M. J. , Richardson, R. S. , & Amann, M. (2020). The muscle reflex and chemoreflex interaction: Ventilatory implications for the exercising human. Journal of Applied Physiology, 129(4), 691–700. 32816637 10.1152/japplphysiol.00449.2020 PMC7654695

[eph13506-bib-0087] Wang, H. J. , Li, Y. L. , Gao, L. , Zucker, I. H. , & Wang, W. (2010). Alteration in skeletal muscle afferents in rats with chronic heart failure. The Journal of Physiology, 588(Pt 24), 5033–5047. 21041525 10.1113/jphysiol.2010.199562 PMC3036195

[eph13506-bib-0088] Warner, M. M. , & Mitchell, G. S. (1991). Role of catecholamines and beta‐receptors in ventilatory response during hypoxic exercise. Respiration Physiology, 85(1), 41–53. 1658899 10.1016/0034-5687(91)90005-4

[eph13506-bib-0089] Weil, J. V. , Byrne‐Quinn, E. , Sodal, I. E. , Kline, J. S. , McCullough, R. E. , & Filley, G. F. (1972). Augmentation of chemosensitivity during mild exercise in normal man. Journal of Applied Physiology, 33(6), 813–819. 4643864 10.1152/jappl.1972.33.6.813

[eph13506-bib-0090] Williamson, J. W. , McColl, R. , Mathews, D. , Mitchell, J. H. , Raven, P. B. , & Morgan, W. P. (2002). Brain activation by central command during actual and imagined handgrip under hypnosis. Journal of Applied Physiology, 92(3), 1317–1324. 11842073 10.1152/japplphysiol.00939.2001

[eph13506-bib-0091] Zera, T. , Moraes, D. J. A. , da Silva, M. P. , Fisher, J. P. , & Paton, J. F. R. (2019). The logic of carotid body connectivity to the brain. Physiology, 34(4), 264–282. 31165684 10.1152/physiol.00057.2018

